# Tuberculosis in adult migrants in Europe: a TBnet consensus statement

**DOI:** 10.1183/13993003.01612-2024

**Published:** 2025-03-06

**Authors:** Heinke Kunst, Berit Lange, Olga Hovardovska, Annabelle Bockey, Dominik Zenner, Aase B. Andersen, Sally Hargreaves, Manish Pareek, Jon S. Friedland, Christian Wejse, Graham Bothamley, Lorenzo Guglielmetti, Dumitru Chesov, Simon Tiberi, Alberto Matteelli, Anna M. Mandalakas, Jan Heyckendorf, Johannes Eimer, Akanksha Malhotra, Javier Zamora, Anca Vasiliu, Christoph Lange, for the TBnet

**Affiliations:** 1 Blizard Institute, Barts and The London School of Medicine and Dentistry, Queen Mary University of London, London, UK; 2 Department of Epidemiology, Helmholtz Centre for Infection Research, Braunschweig, Germany; 3 German Center for Infection Research, TI BBD, Braunschweig, Germany; 4 PhD Programme Epidemiology Braunschweig-Hannover, Braunschweig, Germany; 5 Wolfson Institute of Population Health, Barts and The London School of Medicine and Dentistry, Queen Mary University of London, London, UK; 6 Infection and Population Health Department, Institute for Global Health, University College London, London, UK; 7 Dept of Infectious Diseases, Copenhagen University Hospital Rigshospitalet, Copenhagen, Denmark; 8 The Migrant Health Research Group, Institute for Infection and Immunity, City St George's, University of London, and Lancet Migration European Regional Hub, London, UK; 9 Department of Respiratory Sciences, University of Leicester, Leicester, UK; 10 Development Centre for Population Health, University of Leicester, Leicester, UK; 11 Department of Infectious Diseases, Aarhus University Hospital, Aarhus, Denmark; 12 Homerton University Hospital, London, UK; 13 Faculty of Infectious and Tropical Diseases, London School of Hygiene and Tropical Medicine, London, UK; 14 Sorbonne Université, INSERM, U1135, Centre d'Immunologie et des Maladies Infectieuses, Cimi-Paris, APHP Sorbonne Université, Hôpital Pitié-Salpêtrière, Laboratoire de Bactériologie-Hygiène, Centre National de Référence des Mycobactéries et de la Résistance des Mycobactéries aux Antituberculeux, Paris, France; 15 Department of Pneumology and Allergology, Nicolae Testemitanu State University of Medicine and Pharmacy, Division of Clinical Infectious Diseases, Chisinau, Moldova; 16 Clinical Infectious Diseases, Research Center Borstel, Leibniz Lung Center, Borstel, Germany; 17 Clinic of Infectious and Tropical Diseases, WHO Collaborating Centre for TB prevention, Department of Clinical and Experimental Medicine, University of Brescia, Brescia, Italy; 18 Baylor College of Medicine and Texas Children's Hospital, Global TB Program, Houston, TX, USA; 19 Clinical Tuberculosis Unit, German Center for Infection Research (DZIF), Hamburg-Lübeck-Borstel-Riems, Germany; 20 Leibniz Lung Clinic, Department of Internal Medicine I, University Clinic Schleswig-Holstein Campus Kiel, Kiel, Germany; 21 Division of Infectious Diseases and Tropical Medicine, Department of Internal Medicine 4 – Pneumology, Kepler University Hospital and Medical Faculty, Johannes Kepler University, Linz, Austria; 22 Clinical Biostatistics Unit, Hospital Ramon y Cajal (IRYCIS, CIBERESP), Madrid, Spain; 23 Respiratory Medicine and International Health, University of Lübeck, Lübeck, Germany; 24 H. Kunst and B. Lange are joint first authors; 25 A. Vasiliu and C. Lange are joint last authors

## Abstract

**Introduction:**

Global migration has increased in recent decades owing to war, conflict, persecution and natural disasters, but also secondary to increased opportunities related to work or study. Migrants’ risk of tuberculosis (TB) differs depending on migration, socioeconomic status, mode of travel and TB risk in transit, TB incidence and healthcare provision in country of origin. Despite advances in TB care for migrants and new treatment strategies, decisions for managing migrants at risk of TB often rely on expert opinions, rather than clinical evidence.

**Methods:**

A systematic literature search was conducted, studies were mapped to different recommendation groups and included studies were synthesised by meta-analysis where appropriate. Current evidence on the diagnosis of active TB in migrants entering the European Union/European Economic Area and UK, including clinical presentation and diagnostic delay, treatment outcomes of drug-susceptible TB, prevalence, and treatment outcomes of multidrug-resistant/rifampicin-resistant TB and TB/HIV co-infection, was summarised. A consensus process was used based on the evidence.

**Results:**

We documented that migrants had higher vulnerability for TB, including an increased risk of extrapulmonary TB, multidrug-resistant/rifampicin-resistant TB, TB/HIV co-infection and worse TB treatment outcomes compared to host populations. Consensus recommendations include screening migrants for TB/latent TB infection according to country data, a minimal package for TB care in drug-susceptible and multidrug-resistant/rifampicin-resistant TB, implementation of migrant-sensitive strategies and free healthcare and preventive treatment for migrants with HIV co-infection.

**Conclusion:**

Dedicated care for TB prevention and treatment in migrant populations within the European Union/European Economic Area and UK is essential.

## Shareable abstract

**Migrant populations entering Europe have poorer TB outcomes than native populations. As TB case numbers rise in Europe, we need robust strategies to strengthen screening, rapid diagnosis and treatment in these hard-to-reach populations.** https://bit.ly/3OFoz3Y

## Graphical abstract

**Figure:**
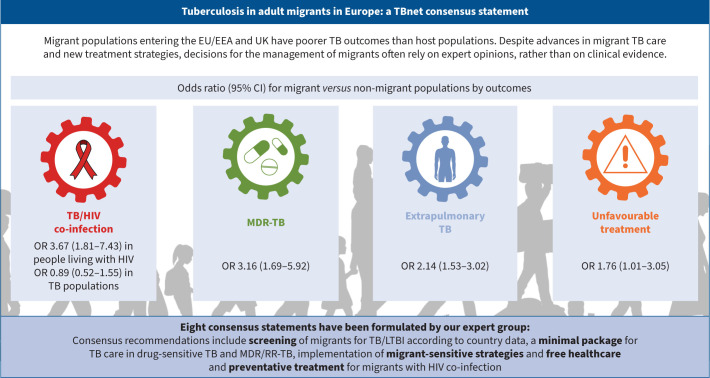
Overview of the study. TB: tuberculosis; MDR: multidrug-resistant; LTBI: latent tuberculosis infection; RR: rifampicin-resistant.

## Introduction

Migration is a significant global phenomenon; currently, around 281 million people (3.6% of the global population) live outside their country of origin and are defined as international migrants [1]. Reasons, circumstances and routes of international migration vary considerably. Causes of migration include conflict and war, persecution, natural disasters, work opportunities and study [1]. Tuberculosis (TB) is an infectious disease that commonly affects the lungs. TB is the leading cause of death from a single bacterial infectious agent worldwide [2]. Migrants may have a higher risk of TB than native-born individuals [3], related to factors such as TB prevalence in their country of origin, migration route, living conditions in the host country, time of residence in the host country and frequency of visiting their country of origin [3–6]. Studies have shown significant differences in the yield of TB screening in migrant workers compared with refugees and asylum seekers, with the highest TB prevalence among the more vulnerable migrants [6]. At the end of 2022, 108.4 million people worldwide were forcibly displaced as a result of persecution, conflict, violence or human rights violations. In 2022, over one in every 74 people globally have been forced to flee their homes [7]. In 2023, it was estimated that over half of all refugees under the United Nations High Commissioner for Refugees mandate and other people in need of international protection come from just three countries, Syria (6.7 million), Afghanistan (6.1 million) and Ukraine (5.9 million) [8].

In 2020, there were more than 9000 foreign-born individuals developing TB in the European Union (EU)/European Economic Area (EEA) [9]. The number of Ukranians who were notified with TB in the EU/EEA increased from 164 (27 with multidrug-resistant (MDR)/rifampicin-resistant (RR) TB) to 780 (194 with MDR/RR-TB) between 2020 and 2022, indicating substantial changes in the TB epidemiology in the EU/EEA in recent times [10].

TB programmes for migrants have been a key priority of host countries in the EU/EEA and the UK [11]. Migrant-sensitive strategies, *e.g.* availability of interpreters and language-appropriate written materials, healthcare provider training in culture-sensitive issues, health education of migrants and social support, are thought to be essential for migrant care [12]. Migrants should have unhindered access to prevention, diagnostics and TB treatment integrated with other health services, equal to that of the native population.

We conducted a systematic review to investigate if there are different outcomes among migrants with regards to TB diagnosis, TB treatment, MDR/RR-TB and TB/HIV co-infection compared to non-migrants, including use of migrant-sensitive strategies *versus* no strategies in migrant care. In a modified Delphi process, we developed consensus guidance for early diagnosis and effective treatment of drug-susceptible TB, MDR/RR-TB and TB/HIV co-infection in migrants in the EU/EEA and UK.

## Methods

### Definition of a migrant

The definition of a migrant followed the recommendation of the European Centres of Disease Control and Prevention (ECDC): “Any individual who lives in a country temporarily or permanently away from his or her usual place of residence for at least a year” [11]. We defined migrant types according to a modified classification of migrant status of the International Organisation of Migration (supplementary table S1).

### Systematic review

#### Expert groups subject

Expert panel groups were convened on four topics for migrants entering the EU/EEA and UK. These were 1) TB diagnosis, 2) TB treatment, 3) MDR/RR-TB and 4) TB/HIV co-infection.

#### Evidence mapping

The Preferred Reporting Items for Systematic reviews and Meta-Analyses (PRISMA) systematic review methodology [13] and the Reporting Items for practice Guidelines in HealThcare (RIGHT) statement for reporting recommendations [14] were followed. The Population, Intervention, Comparator, Outcome (PICO) model was used, with the PICOs designed by the methods groups (B. Lange, C. Lange, H. Kunst) and, after revisions, agreed by all expert chapter groups. The systematic review includes as a population migrants and non-migrants managed at clinical TB centres in the EU/EAA and UK (supplementary table S2). The “Diagnosis of active TB” chapter includes the spectrum of clinical presentation and delay in diagnosis in migrants *versus* non-migrants. Diagnostic delay is divided into patient delay and health system delay [15]. The “Treatment of active TB” chapter includes an evaluation of the treatment of drug-susceptible TB in migrants *versus* non-migrants and management issues including use of migrant-sensitive *versus* no migrant-sensitive strategies. The “MDR/RR-TB and migration” chapter includes an evaluation of the prevalence of MDR/RR-TB compared to drug-susceptible TB in migrants *versus* non-migrants and MDR/RR treatment outcomes in migrants *versus* non-migrants. In addition, management issues, including use of migrant-sensitive *versus* no migrant-sensitive strategies in MDR/RR-TB, are evaluated. The “TB/HIV co-infection and migration” chapter includes an evaluation of the diagnosis and management of TB/HIV co-infection in migrants *versus* non-migrants, the prevalence of TB/HIV co-infection in migrants *versus* non-migrants and TB treatment outcomes in migrants *versus* non-migrants.

The systematic review was registered with PROSPERO (CRD42018074338). OVID MEDLINE, EBSCO CINAHL and Web of Science databases were searched using the subject keywords and Medical Subject Heading (MeSH) terms for “TB” and “migrant” (search strategy in supplementary material).

The search was restricted to the English language and included all articles from inception until April 2024 (supplementary figure S1). We included observational cohort studies including non-controlled cohort studies and interventional studies. References of review papers and relevant articles were also searched. In addition, reference lists of the included studies were searched for eligible articles.

After screening publications from a literature search and subsequent selection by title, abstract or full-text, each publication was screened by two of four reviewers (A. Bockey, O. Hovardovska, B. Lange, H. Kunst). For dissident opinions regarding article inclusion among two reviewers, consensus was sought from the other two reviewers. Evidence bundles were distributed to the expert groups for each chapter with the request to check for possible missing relevant studies.

#### Data extraction in chapter groups

Data on study characteristics and addressing the PICOs were subsequently extracted for each topic by expert groups. Where evidence from the literature was limited, expert consensus on best evidence was used and agreed upon by the group. Additional references provided by expert groups or external experts were included. When data from the published articles were not clear enough, we contacted the authors for clarification.

#### Data synthesis and analysis

Studies were synthesised by meta-analysis where appropriate; otherwise, narrative synthesis was used. Where available, we pooled risk estimates of odds ratios (ORs) and relative risks through meta-analysis using the R software with the *metafor* package [16]. We included pooled estimates irrespective of study design. The point estimate was calculated using random effects models to account for potential variations in effect sizes across studies. The standard error and 95% confidence intervals of risk estimates were calculated from data reported in each paper. Heterogeneity was assessed visually and using both I^2^ and τ^2^ measures.

#### Quality assessment

The quality of the studies was assessed using the Newcastle-Ottawa Scale [17]. We considered studies to have a low risk of bias if they scored four stars for selection, two for comparability and three for ascertainment of outcomes. Studies with two or three stars for selection, one for comparability and two for outcome ascertainment were regarded as having a medium risk of bias, and any study scoring one star for selection or outcome ascertainment, or zero for any of the three categories was considered to have a high risk of bias. Two reviewers independently conducted the quality assessment using the Newcastle-Ottawa Scale (A. Malhotra, H. Kunst) and any discrepancies were resolved with the involvement of a third reviewer.

### Consensus statements

The consensus recommendations were based on the evidence found and conducted through a modified Delphi process according to previously used methodology [18–20]. We followed five steps for drafting final consensus statements.

Step 1: Preliminary proposals for five key recommendations were drafted by the coordinating authors. Chapter leaders were asked to provide alternative statements following consultation with the co-authors of their chapter.

Step 2: Alternative statements were collected by the coordinating authors.

Step 3: Chapter leaders were asked to select one preferred statement among the alternative statements following consultation with the co-authors of their chapter.

Step 4: For each recommendation, the statement that received most votes was selected for inclusion in the manuscript.

Step 5: All co-authors were asked to indicate their agreement, disagreement or whether they preferred to abstain from a decision on a recommendation. Results of the decisions are indicated at the end of each consensus statement.

## Results

### Evidence mapping

A total of 7420 records were identified and screened by four reviewers (A. Bockey, B. Lange, H. Kunst, O. Hovardovska). We identified 492 studies that were relevant to individual chapter groups. These were put together as evidence bundles and shared with the authors (supplementary figure S1). Of 492 studies, 113 studies were included in the narrative review and, from those, 49 studies were included in the meta-analysis (supplementary tables S3, S5, S7–S9).

### Diagnosis of active TB in migrants including the spectrum of clinical presentation and diagnostic delay

**FIGURE 1:**
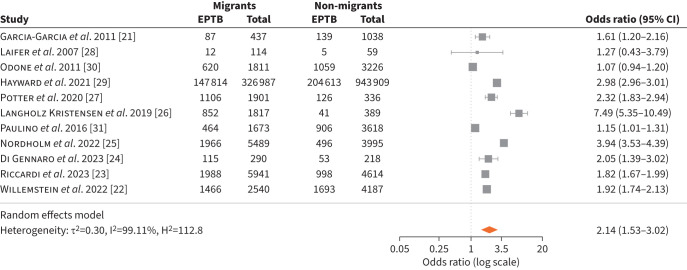
Diagnosis of active tuberculosis (TB): extrapulmonary TB (EPTB) *versus* pulmonary TB in migrants *versus* non-migrants.

We included 11 studies addressing the clinical presentation of TB in migrants compared to non-migrants [21–31]. Pulmonary TB was the predominant presentation at diagnosis in all but two of the studies [26, 27]. Overall, migrants were more likely to be diagnosed with extrapulmonary TB (EPTB) than non-migrants (OR 2.14, 95% CI 1.53–3.02) (figure 1). There was significant heterogeneity between studies. The largest study based on data from the ECDC European Surveillance System (TESSy) found an OR of 2.98 (95% CI 2.96–3.01) for EPTB in migrants compared to non-migrants (study period 1995–2017; n=1 270 896) [29]. A lower proportion of EPTB was seen in Eastern Europe (17.4%; 98 656 of 566 170) and Southern Europe (29.6%, 62 481 of 210 828) compared with Western Europe (35.7%, 89 498 of 250 517) and Northern Europe (41.8%, 101 792 of 243 381). Migrants from South-East Asia and Sub-Saharan Africa were at highest risk of EPTB, with 62.0% (55 401 of 89 353) and 54.5% (38 327 of 70 378) of cases, respectively [29]. To determine if our findings were solely explained by the large ECDC study, we conducted a sensitivity analysis excluding this study from the meta-analysis, which demonstrated no significant impact on the results (supplementary figure S2).

A separate analysis using surveillance data collected by the ECDC system TESSy over a shorter period (2003–2014) examined the risk for specific organ presentations according to the region of origin of migrants. ORs varied considerably between and within regions of origin, with the highest ORs for lymphatic, osteoarticular and peritoneal/digestive EPTB in patients from the Indian subcontinent (OR range of 6.4–8.8) [32]. The majority of studies did not provide data on migrant status, *i.e.* refugee *versus* migrant worker. A study from Denmark reported a similar risk of EPTB among migrant subcategories. EPTB was present in 852 of a total of 1841 migrants (46.3%); the rates of EPTB in migrant subcategories ranged from 40.2% to 49.5%. The study showed a significantly higher risk of EPTB in migrants compared to the other studies included in the meta-analysis (OR 7.49, 95% CI 5.35–10.49) [26].

Seven studies on diagnostic delay were identified [15, 27, 33–37]. Patient delay has been defined as the time delay from symptom onset to first consultation; and health system delay from time of first consultation to diagnosis and/or treatment initiation [15]. Three studies evaluated patient and health system delay in migrants *versus* non-migrants (supplementary table S4). All three studies showed that patient delay was higher in migrants than non-migrants, whereas health system delay was shorter in migrants than non-migrants [15, 35, 37]. Risk factors for patient delay among migrants included perceived stigma, distance to the healthcare provider and healthcare cost. Out of the three studies, two included patients with pulmonary TB and EPTB, and one study from Denmark only included cases with EPTB [35]. A study from the UK demonstrated an increase in the proportion of patients with any delay above the median time from symptom onset to treatment following the introduction of a migrant cost recovery programme requesting migrants to pay for their care. The difference reached statistical significance for migrants, but the same trends towards a prolonged delay were observed in UK-born patients [27]. An Italian single-centre study showed a diagnostic delay in migrants from high TB incidence countries that was almost twice as high as that for native-born patients (n=48, median 153 days (IQR 7–556 days) *versus* 88 days (IQR 12–28 days)) [36]. A Spanish study including only migrants reported on patient and health service delays and found that delays varied according to country of origin and education level. Delays were significantly increased in EPTB compared to pulmonary TB [34]. A UK study investigated patient and health system delays in pulmonary TB patients and found that the shorter the time since entry to the UK, the longer the health system delay [33].

### Treatment of active TB in migrants and management issues

**FIGURE 2:**
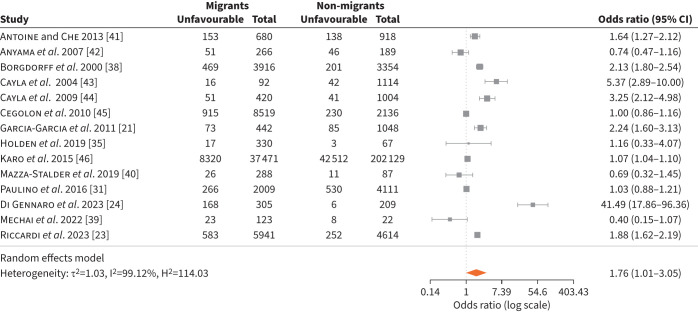
Unfavourable tuberculosis treatment outcomes in migrants *versus* non-migrants.

We identified 14 studies [21, 23, 24, 31, 35, 38–46] reporting on treatment outcomes in migrants *versus* non-migrants that we included in the meta-analysis (figure 2). These studies included 60 802 migrants with TB and 221 002 non-migrants with TB. There were three studies from Spain [21, 43, 44], two from the UK [42, 45], Italy [23, 24] and France [39, 41], one from the Netherlands [38], Denmark [35], Switzerland [40] and Portugal [31] and one study included several European countries [46]. Studies used different definitions of unfavourable TB treatment outcomes including those for treatment default, treatment failure and loss to follow-up. Unfavourable treatment outcomes were higher in migrants than non-migrants, with a pooled OR of 1.76 (95% CI 1.01–3.05) (figure 2). One study from France evaluating TB mortality among migrants compared to non-migrants showed a higher mortality for the native population than migrants (HR 8.0, 95% CI 3.0–21.5) [47].

Included studies did not report on subcategories of migrants and therefore the analysis could not be stratified by migrant status. We could not identify any studies comparing the use of migrant-sensitive strategies *versus* no strategies with regards to treatment adherence and completion.

### MRD/RR-TB and migration

**FIGURE 3:**
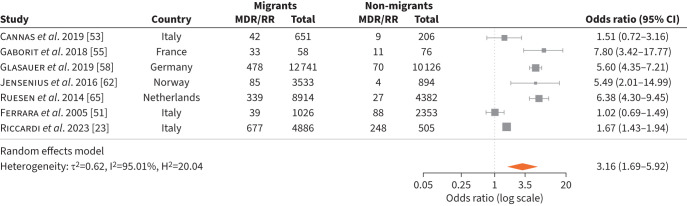
Multidrug-resistant/rifampicin-resistant tuberculosis (MDR/RR-TB) rates in migrants.

We found 25 studies reporting on MDR/RR-TB prevalence in migrants in the EU/EAA and UK [23, 24, 48–70]. There were six studies from Italy [23, 24, 51, 53, 54, 66], three from the Netherlands [60, 65, 67] and France [49, 50, 55], two from Spain [56, 69], Portugal [52, 64] and Greece [61, 63], and one from Belgium [70], Czech Republic [48], Germany [58], Finland [71], Norway [62], Sweden [57] and Switzerland [59]. The rates of MDR/RR-TB varied according to geographical area and study period (supplementary tables S6 and S7). For example, studies from Spain and Portugal showed lower rates of MDR/RR-TB among migrants compared to studies from the Netherlands. Seven of the 25 studies [23, 51, 53, 55, 58, 62, 65] compared rates of MDR/RR-TB and drug-susceptible disease in migrants and non-migrants, showing that MDR/RR-TB was more common in migrants than non-migrants, with a pooled OR of 3.16 (95% CI 1.69–5.92) (figure 3).

In a Portuguese study, treatment outcomes in MDR/RR-TB were more likely to be unfavourable in migrants than non-migrants (OR 1.78, 95% CI 1.04 –3.05) [52]. Similarly, a UK study showed better treatment outcomes in non-migrants than migrants, with an adjusted OR of 0.45 (95% CI 0.10–1.92) [72]. A study from Italy suggested that migrants with MDR/RR-TB were more likely to default from treatment than non-migrants, with 12 of 39 (30.7%) *versus* nine of 88 (10.2%) defaulting, respectively [51]. A Czech study among patients with MDR/RR-TB showed that 15 of 17 migrants (88.2%) compared to 21 of 33 non-migrants (63.6%) achieved cure as a treatment outcome [48]. Another study from Portugal did not show a significant difference in mortality in migrants with MDR/RR-TB compared to non-migrants [64].

### TB/HIV co-infection and migration

A total of 20 studies reported on TB/HIV co-infection prevalence and treatment outcomes in migrants compared to non-migrants. Of these, 17 studies [23, 24, 31, 73–86] were included in the meta-analysis on TB/HIV co-infection prevalence and three studies [81, 85, 87] reported on treatment outcomes of TB/HIV co-infection, with one of them not being included in the meta-analysis [87]. Out of the 17 studies, 10 [23, 24, 31, 73, 74, 77–79, 83, 84] reported on TB/HIV co-infection rates in populations with TB, and the remaining seven studies [75, 76, 80–82, 85, 86] reported on people living with HIV.

**FIGURE 4:**
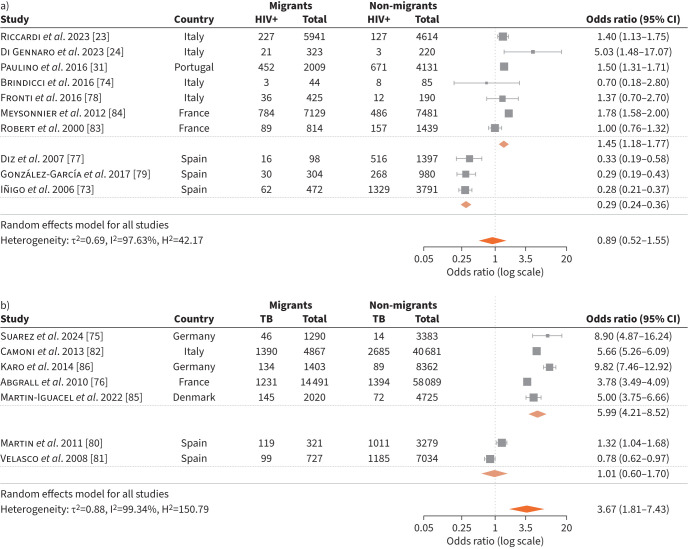
Tuberculosis (TB)/HIV co-infection in a) TB populations and b) people living with HIV.

Among the 10 studies conducted in TB populations, there were four from Italy [23, 24, 74, 78], three from Spain [73, 77, 79], two from France [83, 84] and one from Portugal [31]. Studies showed significant heterogeneity; TB/HIV co-infection prevalence in migrants *versus* non-migrants varied according to the country where the study was conducted. However, the overall risk of TB/HIV co-infection was lower in migrants than non-migrants, with a pooled OR of 0.89 (95% CI 0.52–1.55) (figure 4a). Subgroup analysis excluding the three studies from Spain [73, 77, 79] showed higher rates of co-infection in migrants than non-migrants, with a pooled OR of 1.45 (95% CI 1.18–1.77). The three Spanish studies showed lower rates of co-infection in migrants than non-migrants, with a pooled OR of 0.29 (95% CI 0.24–0.36). Two of these studies [73, 79] reported significantly higher rates of intravenous drug abuse among non-migrants than migrants (17% *versus* 1%, and 27.9% *versus* 5.3%, respectively).

Three studies that could not be included in the meta-analysis showed higher rates of TB/HIV co-infection in migrants than non-migrants. In a large cohort study from France (n=14 610), migrants had a higher rate of co-infection than non-migrants (11.0% *versus* 6.5%; p=0.001) [84]. Over 10% of TB patients in a UK study were reported to have TB/HIV co-infection [88]. Patients with co-infection were more likely to be migrants than patients who were not co-infected or who were not tested for HIV (OR 2.6, 95% CI 2.6–4.4; p<0.0001 on univariate analysis). An analysis of risk factors from another UK study showed that the likelihood of TB/HIV co-infection was almost eight times higher among migrants than non-migrants (OR 7.9, 95% CI 4.5–14.4; p<0.00001) [89].

The seven studies [75, 76, 80–82, 85, 86] that estimated TB rates in people living with HIV also showed significant heterogeneity but overall odds of having co-infection were higher in migrants than non-migrants, with a pooled OR of 3.67 (95% CI 1.81–7.43). Out of the seven studies, there were two studies from Spain [80, 81] and Germany [75, 86] and one from Denmark [85], France [76] and Italy [82]. Rates of TB/HIV co-infection in migrants and non-migrants varied across countries. An Italian study showed that the proportion of migrants among co-infected cases increased from 10.8% in 1993 to 64.6% in 2010 (chi-squared test for trend p<0.001) [82].

Subgroup analysis excluding the two Spanish studies [80, 81] showed a higher OR of TB/HIV co-infection in migrants *versus* non-migrants (OR 5.99, 95% CI 4.21–8.52) (figure 4b). Similar to the TB population cohort, one study from Spain showed a higher prevalence of TB among people living with HIV in non-migrants compared to migrants; however, intravenous drug abuse in this study was significantly more prevalent in non-migrants than migrants (83.5% *versus* 26%; p<0.001). Nevertheless, differences in the number of active TB episodes per patient, the type of TB, CD4^+^ T-lymphocyte count and viral load were not significantly different between migrants and non-migrants [81]. The other study from Spain did not report on drug abuse and showed higher rates of AIDS and TB among migrants than non-migrants (37.1% *versus* 30.8%, respectively; p=0.02) [80].

TB treatment outcomes in TB/HIV co-infection were assessed in three studies reporting on TB in HIV populations [81, 85, 87]. Non-migrants had an increased risk of death in a Danish study with an adjusted mortality risk of 2.3 (95% CI 1.3–4.3) [85]. Similarly, in a study from Spain, better survival was associated with being a migrant (median 8.7 *versus* 5.4 years; p<0.001); the overall mortality rate was 41.7%, with a mortality rate of 17.2% for migrants and 45.2% for non-migrants (p<0.001) [81]. In contrast, a study from Italy (84 out of 246 were migrants) showed that migrant status was associated with a three-fold increase in the risk of unfavourable treatment outcomes (OR 3.4, 95% CI 1.4–8.3; p=0.008) [87].

### Quality assessment

In the risk of bias quality assessment, 18 out of 49 studies were assessed to be at low risk of bias, 27 studies were at medium risk of bias and four studies were at high risk of bias (supplementary figure S3).

### Consensus recommendations

Based on the evidence reviewed (table 1), eight consensus questions were developed, and the recommendations of the expert groups are detailed below.

**TABLE 1:** TB outcomes of migrants *versus* non-migrants

TB outcomes	Studies (n)	Migrants with events (n/N)	Non-migrants with events (n/N)	Odds ratio (95% CI)	I^2^ (%)^#^
**Diagnosis**					
EPTB	11	156 490/349 000	210 129/965 589	2.14 (1.53–3.02)	99.1
**Treatment**					
Unfavourable treatment outcome	14	11 131/60 802	44 105/221 002	1.76 (1.01–3.05)	99.1
**Drug resistance**					
MDR/RR-TB	7	1693/31 809	457/18 542	3.16 (1.69–5.92)	95.0
**TB/HIV co-infection**					
In TB populations	10	1720/17 599	3577/24 328	0.89 (0.52–1.55)	97.6
In HIV populations	7	3164/25 119	6450/125 553	3.67 (1.81–7.43)	99.3

TB: tuberculosis; EPTB: extrapulmonary tuberculosis; MDR/RR: multidrug-resistant/rifampicin-resistant. ^#^: I^2^ values >50% show significant heterogeneity among the studies.

#### 1) Should all migrants from countries of origin with a TB prevalence of >100 per 100 000 as estimated by the World Health Organization (WHO) be screened for active TB and latent TB infection (LTBI) with *Mycobacterium tuberculosis* when entering the EU/EEA and UK?

All migrants from countries of origin with a TB prevalence of >100 per 100 000 as estimated by the WHO should be screened for active TB and latent infection with *M. tuberculosis* when entering the EU/EEA and UK. However, screening policies should additionally be informed by local data.

Agreed: n=16 (80%); disagreed: n=3 (15%); abstained: n=1 (5%)

#### 2) If screening for active TB or latent infection with *M. tuberculosis* was not performed when a migrant at risk for TB entered the EU/EEA or UK, how long after entering the EU/EEA or UK should screening still be performed?

All migrants at risk for active TB or latent infection with *M. tuberculosis* regardless of whether they were screened in their country of origin or when entering the EU/EAA or UK should be screened for TB or LTBI within the first 2 years after entering the EU/EEA or UK.

Agreed: n=15 (75%); disagreed: n=4 (20%); abstained: n=1 (5%)

#### 3) What is the minimal package that should be offered to ensure treatment adherence and completion in migrants to the EU/EEA and UK?

The minimal package for TB care for migrants with TB in any country of the EU/EEA and UK should include access to an interpreter if required, information material available in different languages and healthcare staff trained in migrant- and culture-sensitive issues. Screening for blood-borne viruses, an updated vaccination schedule for vaccine-preventable diseases and advice on access to health and mental health services and antenatal care should be included in a general package for migrants, including access to free primary care.

Agreed: n=19 (95%); disagreed: n=0 (0%); abstained n=1 (5%)

#### 4) What should be the management of migrant TB patients in the EU/EEA and UK who do not have healthcare coverage in the arriving country?

Migrants with active TB in the EU/EEA and UK who do not have the benefit of health insurance should receive TB treatment free of charge in the host country in order to reduce the risk of non-completion of treatment, loss to follow-up and *M. tuberculosis* transmission. Migrants should have access to free healthcare inclusive of diagnostics and treatments of diseases of public health concern such as but not exclusive to TB and HIV. Migrants should not be deported until treatment has been successfully completed.

Agreed: n=14 (70%); disagreed: n=3 (15%); abstained: n=3 (15%)

#### 5) What is the minimum package that should be offered to migrants with MDR/RR-TB in the EU/EEA and UK?

Migrants who are diagnosed with MDR/RR-TB in the EU/EEA and UK should receive the same comprehensive care as native patients free of charge even if healthcare coverage is not universal as per their local migratory status. Transfer back to the country of origin prior to completion of treatment is generally discouraged owing to the dire consequences of treatment failure on individual prognosis and further development of drug resistance. This applies in particular to patients who migrate with known MDR/RR-TB and/or prior unsuccessful courses of treatment, which may be reflected in challenging second-line drug resistance patterns. Re-culture of biological samples and reassessment of drug-resistance patterns should always be performed even if available from the country of origin. We further recommend local follow-up for relapse-free cure for 12 months in all cases. This should be rigorously supported for medical reasons by migratory authorities in the case of migrants who are otherwise ineligible for asylum or residence.

Agreed: n=16 (80%); disagreed: n=2 (10%); abstained: n=2 (10%)

#### 6) Should all migrants with HIV entering the EU/EAA and UK be given preventive therapy for LTBI regardless of LTBI status?

All migrants with HIV, in whom active TB was ruled out, should have preventive therapy with a short regimen (daily rifapentine and isoniazid if available) regardless of anti-retroviral treatment status and interferon-γ/tuberculin skin test result, because the risk of a false-negative interferon-γ/tuberculin skin test result outweighs the risk of adverse effects from TB preventive treatment.

Agreed: n=15 (75%); disagreed: n=2 (10%); abstained: n=3 (15%)

#### 7) Should screening for EPTB be considered in migrants?

The prevalence of EPTB and frequency of organ manifestations appear to vary between migrants from different regions of origin. There is insufficient evidence to warrant systematic screening. Clinicians should maintain a high index of suspicion regarding extrapulmonary manifestations, especially in migrants from South-East Asia and Sub-Saharan Africa.

Agreed: n=20 (100%); disagreed: n=0 (0%); abstained n=0 (0%)

#### 8) What measures should be undertaken to minimise diagnostic delay in migrants with active TB?

Access to primary care should be made available to migrants in a clear and understandable way, so that they can be reviewed, receive vaccines and boosters, and access health screening and care if required. Diagnostic delay can only be minimised by multimodal approaches. Active case finding and screening, educational campaigns to reduce stigma, low barriers to care and provider training are key elements of a minimisation strategy. Costs for care for TB and HIV should be waivered.

Agreed: n=19 (95%); disagreed: n=0 (0%); abstained n=1 (5%)

## Discussion

Our systematic review highlights that migrants generally access care later and have worse TB outcomes than non-migrants. Overall, migrants were more likely than non-migrants to be diagnosed with EPTB rather than pulmonary TB, but evidence was insufficient to draw conclusions on the risk of EPTB in migrant subgroups. Phylogenetic subgroups of *M. tuberculosis* are known to be associated with different clinical manifestations. For example, Indo-Oceanic and East-African Indian lineages have been previously associated with EPTB, which may explain the higher percentage of EPTB in migrants *versus* non-migrants [90]. The diagnostic delays described in our review occurred at different timepoints and included factors that were patient related (access to healthcare) and health-system related (barriers to care, stigma, delay in diagnosis and treatment) [15]. Patient delay was higher in migrants than non-migrants, whereas health-system delay was shorter in migrants than non-migrants; however, studies were limited. In addition, the studies reporting on patient and health-system delay included insufficient data and a meta-analysis could not be performed.

Unfavourable TB treatment outcomes were more frequent in migrants than in non-migrants but TB outcomes could not be stratified by migrant status. However, there was significant heterogeneity among studies and treatment outcome definitions varied. Some studies only reported on treatment default as an unfavourable treatment outcome in migrants *versus* non-migrants and did not include other unfavourable treatment outcomes by migrant status, such as death, failure, loss to follow-up and unevaluated, as recommended by WHO guidelines [91].

There were no studies evaluating migrant-sensitive strategies although they are essential for adherence and successful treatment outcomes (cure and treatment completion) in migrants [92]. Knowledge, attitudes, perceptions and social vulnerability of migrant patients may affect treatment outcomes [93]. Expectations of treatment can be affected by understanding of healthcare interactions within the community and country of origin. For example, some migrant groups may use traditional medicines from their country of origin as an adjuvant to or instead of standard treatment [94, 95]. Migrant patients may also lack trust in the healthcare system or professionals due to previous negative experiences, affecting engagement with treatment [96–98]. Lack of financial resources, including the inability to afford transportation, can negatively affect engagement with TB services and treatment [99]. Housing issues may adversely affect health among migrants. Social vulnerabilities can also compound distrust in healthcare services [97, 100] and influence treatment adherence. Stigma can be a barrier to TB diagnosis and treatment uptake [98], with the duality of both being a migrant and having TB exacerbating this situation [94, 99]. Clinical teams, through outreach and keyworkers, may develop links to local support and welfare systems and establish funds for patient transport, housing and community support [101]. Health system-related factors include the knowledge, attitude and skills of healthcare providers, and the patient–provider relationship [99]. Healthcare providers may not understand the issues that migrants are facing and often have not received training in culturally sensitive strategies [102, 103]. Approaching patient–provider relationships in a positive and culturally sensitive way is key to facilitating treatment engagement and improving outcomes [96, 104].

Our review highlighted that MDR/RR-TB is more common in migrants than non-migrants and that treatment outcomes are overall worse and treatment default is higher in migrants, but we did not identify any observational or interventional studies reporting on migrant-sensitive clinical care strategies in migrants affected by MDR/RR-TB. We did not perform a meta-analysis on all 25 studies reporting on MDR/RR-TB. Only seven could be included because the other 18 studies did not have data on drug-susceptible TB.

A previous systematic review found that countries in Europe with a low incidence of TB are declaring increasing numbers of migrants from high-incidence countries, which are overrepresented among MDR/RR-TB cases [105]. Data derived from the ECDC (TESSy) for 2015 showed that the notification rate of MDR/RR-TB was 0.01 per 100 000 in the native population and 64-times higher (*i.e.* 0.89 per 100 000) in individuals who have a nationality or place of birth different from the reporting country [106].

Our review showed unfavourable MDR/RR-TB treatment outcomes were more common in migrants. A previous systematic review in migrants with MDR/RR-TB assessing treatment adherence and using treatment outcome as a substitute for adherence concluded that treatment outcomes of MDR/RR-TB in migrants in Europe are not well recorded [107]. Management of MDR/RR-TB, compared to drug-susceptible TB, is challenging owing to the longer duration of therapy required to achieve cure, even though this may change with the advent of short all-oral regimens, and the overall higher rates of adverse events of treatment. This is particularly relevant for migrant populations in which adherence is challenged by high mobility (including across national borders), additional social and behavioural risk factors and uncertain access to healthcare [105]. A “minimum package” has been proposed to ensure that cross-border TB care is provided, including continuity of care and contact investigation, a legal framework to protect migrants’ rights and efficient data transfer across health facilities in different countries [108]. The implementation of these measures is recommended by the ECDC/European Respiratory Society/European Union Standards of TB care [109]. Transnational collaboration and the use of advanced tools for molecular epidemiology, such as next-generation sequencing, allow prompt investigation of multi-country MDR/RR-TB clusters related to migration [110]. In addition, a few studies performed in referral centres in high-resource and low-incidence countries in Europe have demonstrated that high rates of favourable treatment outcomes and satisfactory treatment adherence are possible to obtain in migrants through personalised MDR/RR-TB treatment, social support and culturally informed healthcare [67, 111–113].

Our review showed that TB/HIV co-infection rates and TB outcomes in migrants *versus* non-migrants varied according to country. Although there was a trend for higher TB rates in migrants, there was no significant risk of worse TB treatment outcomes in migrants with TB/HIV co-infection than in non-migrants, but studies were limited. A previous systematic review also highlighted the heterogeneity of treatment outcomes in patients with TB/HIV co-infection in migrants *versus* non-migrants [114].

We used the Delphi process for our consensus statement because the Delphi method gives controlled feedback, is anonymous and a group of experts achieve consensus in a way that fits the research question with a high level of accuracy. There is a risk of expert members dropping out when using a Delphi process but in our consensus approach this did not happen.

The strength of this review is that it represents the most up-to-date collection of data on TB in adult migrants in the EU/EAA and UK (table 1). The heterogeneity noted in our meta-analysis is typical of systematic reviews and highlights the opportunity to standardise future research examining the utility of strategies aiming to address TB in migrants.

### Conclusion

Our systematic review highlights significant disparities in TB outcomes between migrants and non-migrants. Migrants typically seek care later, present with more extrapulmonary forms of TB and experience worse outcomes than non-migrants. Rates of MDR/RR-TB and of TB/HIV co-infection were higher in migrants than non-migrants although this varied across countries. Consensus recommendations include screening of migrants for TB/LTBI according to country data, a minimal package for TB care in drug-susceptible and MDR/RR-TB, implementation of migrant-sensitive strategies and LTBI preventive treatment for migrants with HIV co-infection. It is essential to develop and implement migrant-sensitive care strategies, enhance healthcare provider cultural competence and establish robust support systems to improve TB outcomes in migrant populations.

## Shareable PDF

10.1183/13993003.01612-2024.Shareable1 This PDF extract can be shared freely online.
Shareable PDF ERJ-01612-2024.Shareable

## Supplementary material

**Please note:** supplementary material is not edited by the Editorial Office, and is uploaded as it has been supplied by the author. Supplementary material ERJ-01612-2024.Supplement
