# Supplementary material for: Tuberculosis in adult migrants in Europe: a TBnet consensus statement
Source: Eur Respir J. 2025 Mar 6;65(3):2401612. doi: 10.1183/13993003.01612-2024 (PMC11883149; doi:10.1183/13993003.01612-2024)
Supplement: Supplementary file 1 [file ERJ-01612-2024.Shareable.pdf]

EUROPEAN RESPIRATORY JOURNAL
REVIEW
H. KUNST ET AL.

SHAREABLE PDF

Tuberculosis in adult migrants in Europe: a TBnet consensus statement

Heinke Kunst, Berit Lange, Olga Hovardovska, Annabelle Bockey, Dominik Zenner, Aase B. Andersen, Sally Hargreaves, Manish Pareek, Jon S. Friedland, Christian Wejse, Graham Bothamley, Lorenzo Guglielmetti, Dumitru Chesov, Simon Tiberi, Alberto Matteelli, Anna M. Mandalakas, Jan Heyckendorf, Johannes Eimer, Akanksha Malhotra, Javier Zamora, Anca Vasiliu and Christoph Lange for the TBnet

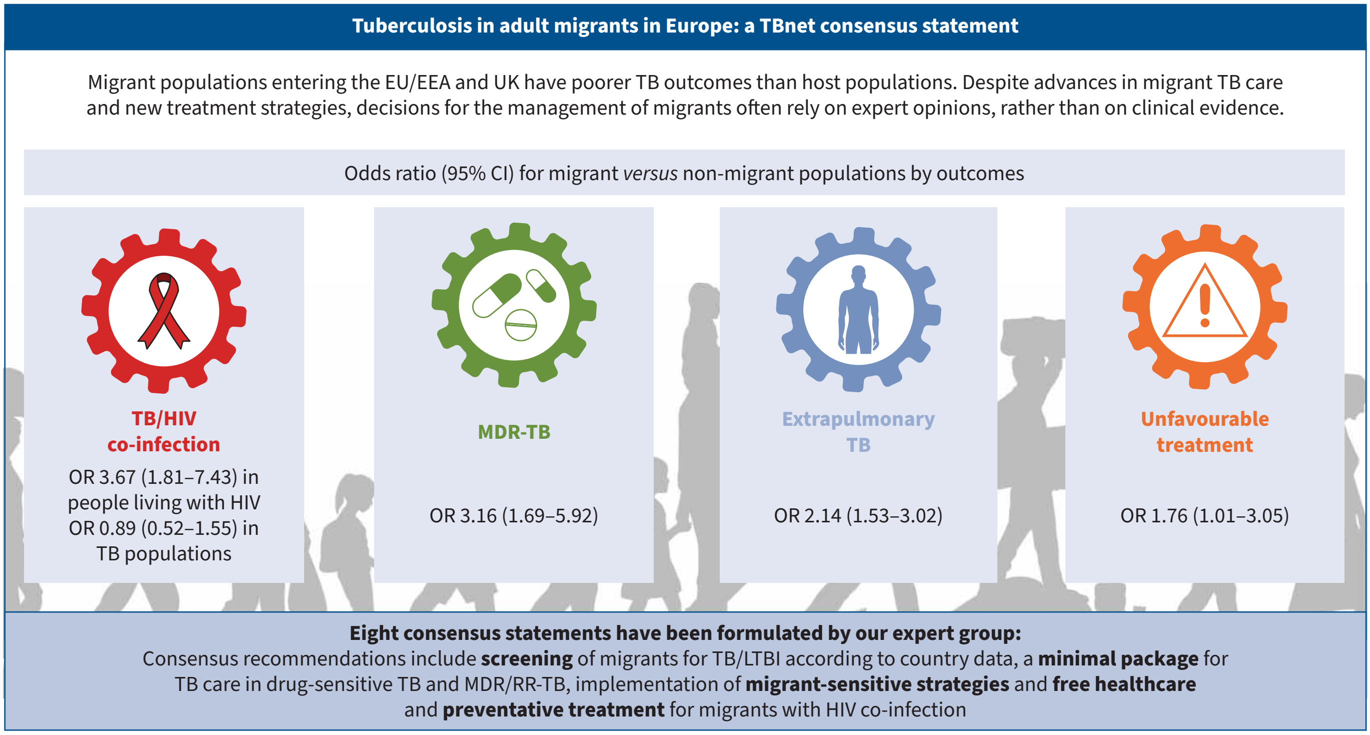

GRAPHICAL ABSTRACT Overview of the study. TB: tuberculosis; MDR: multidrug-resistant; LTBI: latent tuberculosis infection; RR: rifampicin-resistant.

SHAREABLE PDF

Tuberculosis in adult migrants in Europe: a TBnet consensus statement

Heinke Kunst[1,24], Berit Lange[2,3,24], Olga Hovardovska[2,3], Annabelle Bockey[2,3,4], Dominik Zenner[1,5,6], Aase B. Andersen[7], Sally Hargreaves[8], Manish Pareek[9,10], Jon S. Friedland[8], Christian Wejse[11], Graham Bothamley[1,12,13], Lorenzo Guglielmetti[14], Dumitru Chesov[15,16], Simon Tiberi[1], Alberto Matteelli[17], Anna M. Mandalakas[16,18,19], Jan Heyckendorf[20], Johannes Eimer[21], Akanksha Malhotra[1], Javier Zamora[22], Anca Vasiliu[16,18,19,25] and Christoph Lange[16,18,19,23,25] for the TBnet

[1]Blizard Institute, Barts and The London School of Medicine and Dentistry, Queen Mary University of London, London, UK. [2]Department of Epidemiology, Helmholtz Centre for Infection Research, Braunschweig, Germany. [3]German Center for Infection Research, TI BBD, Braunschweig, Germany. [4]PhD Programme Epidemiology Braunschweig-Hannover, Braunschweig, Germany. [5]Wolfson Institute of Population Health, Barts and The London School of Medicine and Dentistry, Queen Mary University of London, London, UK. [6]Infection and Population Health Department, Institute for Global Health, University College London, London, UK. [7]Dept of Infectious Diseases, Copenhagen University Hospital Rigshospitalet, Copenhagen, Denmark. [8]The Migrant Health Research Group, Institute for Infection and Immunity, City St George's, University of London, and Lancet Migration European Regional Hub, London, UK. [9]Department of Respiratory Sciences, University of Leicester, Leicester, UK. [10]Development Centre for Population Health, University of Leicester, Leicester, UK. [11]Department of Infectious Diseases, Aarhus University Hospital, Aarhus, Denmark. [12]Homerton University Hospital, London, UK. [13]Faculty of Infectious and Tropical Diseases, London School of Hygiene and Tropical Medicine, London, UK. [14]Sorbonne Université, INSERM, U1135, Centre d'Immunologie et des Maladies Infectieuses, Cimi-Paris, APHP Sorbonne Université, Hôpital Pitié-Salpêtrière, Laboratoire de Bactériologie-Hygiène, Centre National de Référence des Mycobactéries et de la Résistance des Mycobactéries aux Antituberculeux, Paris, France. [15]Department of Pneumology and Allergology, Nicolae Testemitanu State University of Medicine and Pharmacy, Division of Clinical Infectious Diseases, Chisinau, Moldova. [16]Clinical Infectious Diseases, Research Center Borstel, Leibniz Lung Center, Borstel, Germany. [17]Clinic of Infectious and Tropical Diseases, WHO Collaborating Centre for TB prevention, Department of Clinical and Experimental Medicine, University of Brescia, Brescia, Italy. [18]Baylor College of Medicine and Texas Children's Hospital, Global TB Program, Houston, TX, USA. [19]Clinical Tuberculosis Unit, German Center for Infection Research (DZIF), Hamburg-Lübeck-Borstel-Riems, Germany. [20]Leibniz Lung Clinic, Department of Internal Medicine I, University Clinic Schleswig-Holstein Campus Kiel, Kiel, Germany. [21]Division of Infectious Diseases and Tropical Medicine, Department of Internal Medicine 4 – Pneumology, Kepler University Hospital and Medical Faculty, Johannes Kepler University, Linz, Austria. [22]Clinical Biostatistics Unit, Hospital Ramon y Cajal (IRYCIS, CIBERESP), Madrid, Spain. [23]Respiratory Medicine and International Health, University of Lübeck, Lübeck, Germany. [24]H. Kunst and B. Lange are joint first authors. [25]A. Vasiliu and C. Lange are joint last authors.

Corresponding author: Heinke Kunst (h.kunst@qmul.ac.uk)

Shareable abstract (@ERSpublications)
Migrant populations entering Europe have poorer TB outcomes than native populations. As TB case numbers rise in Europe, we need robust strategies to strengthen screening, rapid diagnosis and treatment in these hard-to-reach populations. https://bit.ly/3OFoz3Y

Cite this article as: Kunst H, Lange B, Hovardovska O, *et al*. Tuberculosis in adult migrants in Europe: a TBnet consensus statement. *Eur Respir J* 2025; 65: 2401612 [DOI: 10.1183/13993003.01612-2024].

This PDF extract can be shared freely online.

Copyright ©The authors 2025.

This version is distributed under the terms of the Creative Commons Attribution Licence 4.0.

Abstract

Introduction Global migration has increased in recent decades owing to war, conflict, persecution and natural disasters, but also secondary to increased opportunities related to work or study. Migrants' risk of tuberculosis (TB) differs depending on migration, socioeconomic status, mode of travel and TB risk in transit, TB incidence and healthcare provision in country of origin. Despite advances in TB care for migrants and new treatment strategies, decisions for managing migrants at risk of TB often rely on expert opinions, rather than clinical evidence.

Methods A systematic literature search was conducted, studies were mapped to different recommendation groups and included studies were synthesised by meta-analysis where appropriate. Current evidence on the

diagnosis of active TB in migrants entering the European Union/European Economic Area and UK, including clinical presentation and diagnostic delay, treatment outcomes of drug-susceptible TB, prevalence, and treatment outcomes of multidrug-resistant/rifampicin-resistant TB and TB/HIV co-infection, was summarised. A consensus process was used based on the evidence.

Results We documented that migrants had higher vulnerability for TB, including an increased risk of extrapulmonary TB, multidrug-resistant/rifampicin-resistant TB, TB/HIV co-infection and worse TB treatment outcomes compared to host populations. Consensus recommendations include screening migrants for TB/latent TB infection according to country data, a minimal package for TB care in drug-susceptible and multidrug-resistant/rifampicin-resistant TB, implementation of migrant-sensitive strategies and free healthcare and preventive treatment for migrants with HIV co-infection.

Conclusion Dedicated care for TB prevention and treatment in migrant populations within the European Union/European Economic Area and UK is essential.
