# Supplementary material for: Tuberculosis in adult migrants in Europe: a TBnet consensus statement
Source: Eur Respir J. 2025 Mar 6;65(3):2401612. doi: 10.1183/13993003.01612-2024 (PMC11883149; doi:10.1183/13993003.01612-2024)
Supplement: Supplementary file 2 [file ERJ-01612-2024.Supplement.pdf]

Supplementary material

Tuberculosis in adult migrants in Europe: a TBnet consensus statement

Authors:

Kunst H[1], Lange B[2,3], Hovardovska O[2,3], Bockey A[2,3;4], Zenner D[1,5,6], Andersen AB[7], Hargreaves S[8], Pareek M[9,10], Friedland JS[8], Wejse C[11], Bothamley G[1,12,13], Guglielmetti L[14], Chesov D[15,16], Tiberi S[1], Matteelli A[17], Mandalakas AM[16,18,19] Heyckendorf J[20], Eimer J[21], Malhotra A[1], Zamora J[22], Vasiliu A[16,18,19], Lange C[16,18,19,23] for the TBnet

Affiliations:

[1] Blizard Institute, Barts and The London School of Medicine and Dentistry, Queen Mary University of London, UK

[2]Department of Epidemiology, Helmholtz Centre for Infection Research, Braunschweig, Germany

[3]German Center for Infection Research, TI BBD, Braunschweig, Germany

[4]PhD Programme Epidemiology Braunschweig-Hannover, Germany

[5]Wolfson Institute of Population Health, Barts and The London School of Medicine and Dentistry, Queen Mary University of London, UK

[6]Infection and Population Health Department, Institute for Global Health, University College London, UK

[7]Dept of Infectious Diseases, Copenhagen University Hospital Rigshospitalet, Copenhagen, Denmark

[8]The Migrant Health Research GROUP, Institute for Infection and Immunity, CITY St George's, University of London; and Lancet Migration European Regional Hub, UK

[9]Department of Respiratory Sciences, University of Leicester, UK

[10]Development Centre for Population Health, University of Leicester, UK

[11]Dept of Infectious Diseases, Aarhus University Hospital, Aarhus, Denmark

[12]Homerton University Hospital, Homerton Row, London, UK

[13]Faculty of Infectious and Tropical Diseases, London School of Hygiene and Tropical Medicine, Keppel Street, London, UK

[14]Sorbonne Université, INSERM, U1135, Centre d'Immunologie et des Maladies Infectieuses, Cimi-Paris; APHP Sorbonne Université, Hôpital Pitié-Salpêtrière, Laboratoire de Bactériologie-Hygiène, Centre National de Référence des Mycobactéries et de la Résistance des Mycobactéries aux Antituberculeux, Paris, France

[15]Department of Pneumology and Allergology, Nicolae Testemitanu State University of Medicine and Pharmacy, Division of Clinical Infectious Diseases, Chisinau, Moldova

[16]Clinical Infectious Diseases, Research Center Borstel, Leibniz Lung Center, Borstel, Germany

[17]Clinic of Infectious and Tropical Diseases, WHO Collaborating Centre for TB prevention, Department of Clinical and Experimental medicine, University of Brescia, Brescia, Italy

[18]Baylor College of Medicine and Texas Children´s Hospital, Global TB Program, Houston, TX, USA

[19]Clinical Tuberculosis Unit, German Center for Infection Research (DZIF), Hamburg-Lübeck-Borstel-Riems, Germany

[20]Leibniz Lung Clinic, Department of Internal Medicine I, University Clinic Schleswig-Holstein Campus Kiel, Germany

[21]Division of Infectious Diseases and Tropical Medicine, Department of Internal Medicine 4 – Pneumology, Kepler University Hospital and Medical Faculty, Johannes Kepler University, Linz, Austria

[22]Clinical Biostatistics Unit. Hospital Ramon y Cajal (IRYCIS, CIBERESP) Madrid, Spain

[23]Respiratory Medicine and International Health, University of Lübeck, Germany

1. Search strategy
2. Table S1 Classification of migrants
3. Figure S1 Prisma flow diagram
4. Figure S2 Sensitivity analysis: Diagnosis of active tuberculosis (extrapulmonary vs pulmonary tuberculosis) studies
5. Figure S3 Newcastle Ottawa scale
6. Table S2 PICOs for each expert chapter group
7. Table S3 Diagnosis of active tuberculosis (extrapulmonary vs pulmonary tuberculosis) studies
8. Table S4 Patient and health system delay studies
9. Table S5 Tuberculosis treatment studies
10. Table S6 Multidrug/rifampicin-resistant tuberculosis studies
11. Table S7 Multidrug/rifampicin-resistant vs drug-susceptible tuberculosis studies
12. Table S8 Tuberculosis /HIV co-infection in tuberculosis populations studies
13. Table S9 Tuberculosis / HIV coinfection in patients living with HIV studies
14. References

Search Strategy

The search strategy included: a specific term for respiratory infections (Infec-SP) a term for refugees, asylees and migrants (Population). Terms were combined using the following schedule:

[(Infec-SP) AND* (Population)

We did not use any search terms specifying included countries or interventions to keep the search strategy as broad as possible.

Web of Science

(tuberculo*) OR (TB) OR (LTBI) OR (latent* NEAR/2 tubercul*) OR (silicotubercul*)

AND

((Refuge*) OR (migrant*) OR (asylum*) OR (immigrant*) OR

(displaced NEAR/2 person*))

OVID

(((tuberculo*.mp.) OR (TB.mp.) OR (LTBI.mp.) OR (latent*.mp. ADJ2 tubercul*.mp.) OR

(silicotubercul*.mp.))

(AND

(((Refuge*.mp.) OR (migrant*.mp.) OR (asylum*.mp.) OR (immigrant*.mp.) OR (displaced.mp. ADJ2 person*.mp.))OR("Refugees"/))

CINAHL

(((tuberculo*) OR (TB) OR (LTBI) OR (latent* N2 tubercul*) OR (silicotubercul*) AND ((Refuge*) OR (migrant*) OR (asylum*) OR (immigrant*) OR (displaced N2 person*))

Supplement Table S1 Classification of migrants

**Migrant status**	**Definition**
Asylum seeker	An individual who is seeking international protection. In countries with individualized procedures, an asylum seeker is someone whose claim has not yet been finally decided on by the country in which he or she has submitted it. Not every asylum seeker will ultimately be recognized as a refugee, but every recognized refugee is initially an asylum seeker.
Documented migrant	A migrant authorized to enter and to stay pursuant to the law of that State or to international agreements to which that State is a party and who is in possession of documents necessary to prove his or her regular status in the country.
Internal migrant	Any person who is moving or has moved within a State for the purpose of establishing a new temporary or permanent residence or because of displacement.
International migrant	Any person who is outside a State of which he or she is a citizen or national, or, in the case of a stateless person, his or her State of birth or habitual residence. The term includes migrants who intend to move permanently or temporarily, and those who move in a regular or documented manner as well as migrants in irregular situations.
Migrant worker	A person who is to be engaged, is engaged or has been engaged in a remunerated activity in a State of which he or she is not a national.
Migrant	An umbrella term, not defined under international law, reflecting the common lay understanding of a person who moves away from his or her place of usual residence, whether within a country or across an international border, temporarily or permanently, and for a variety of reasons. The term includes a number of well-defined legal categories of people, such as migrant workers; persons whose particular types of movements are legally defined, such as smuggled migrants; as well as those whose status or means of movement are not specifically defined under international law, such as international students.
Refugee	A person who, owing to a well-founded fear of persecution for reasons of race, religion, nationality, membership of a particular social group or political opinion, is outside the country of his nationality and is unable or, owing to such fear, is unwilling to avail himself of the protection of that country; or who, not having a nationality and being outside the country of his former habitual residence as a result of such events, is unable or, owing to such fear, is unwilling to return to it .
Undocumented migrant	A non-national who enters or stays in a country without the appropriate documentation.

Source: International Organisation of Migration Glossary on migration https://publications.iom.int/system/files/pdf/iml_34_glossary.pdf

Supplement figure S1 Prisma flow diagram

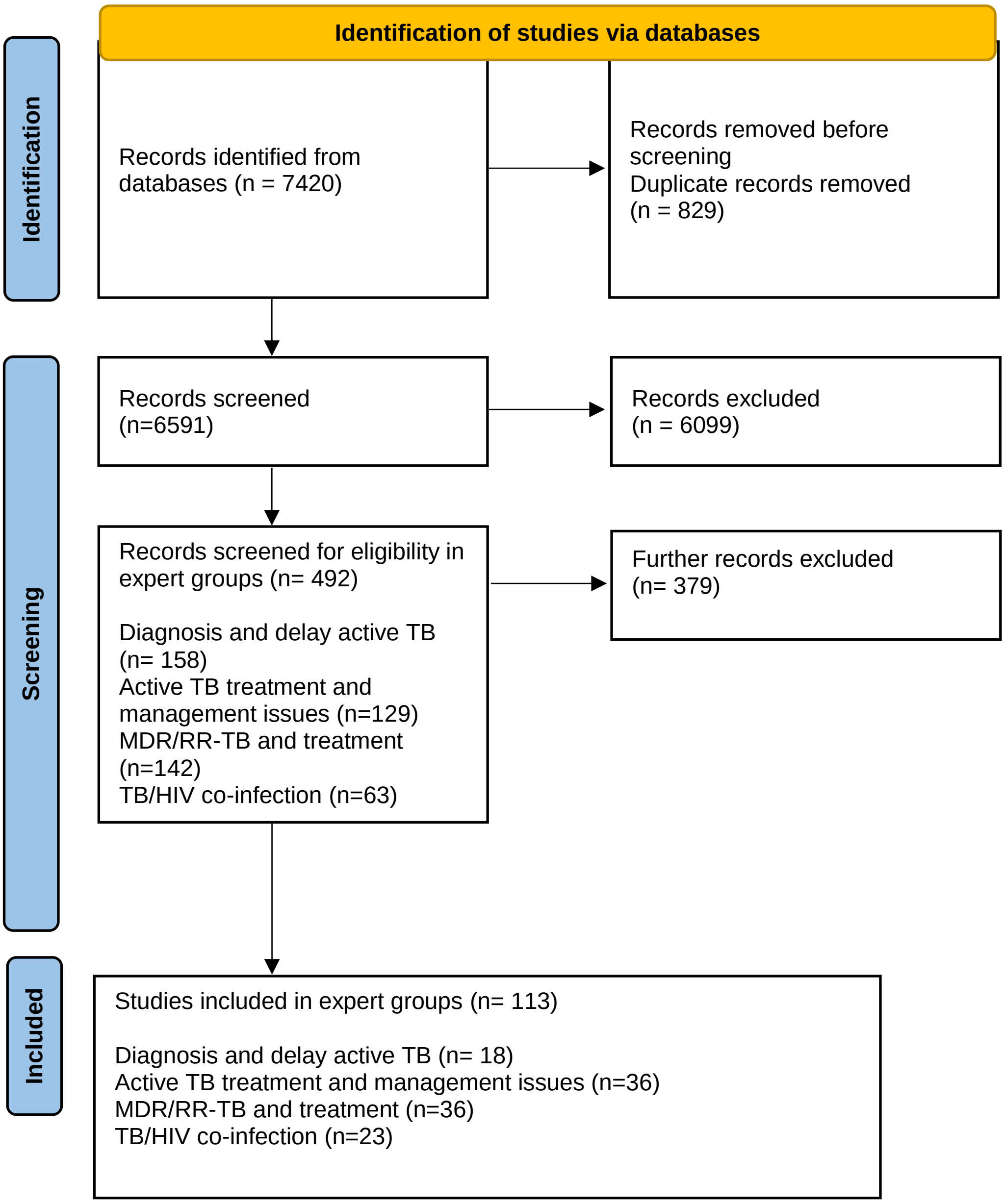

MDR/ RR TB=Multidrug-resistant/ Rifampicin resistant tuberculosis

Supplement figure S2 Sensitivity analysis: Diagnosis of active tuberculosis (extrapulmonary vs pulmonary tuberculosis) studies

	Migrants		Non-migrants		
**Study, year**	**EPTB**	**Total**	**EPTB**	**Total**	**Odds Ratio [95% CI]**
Garcia et al., 2011	87	437	139	1038	1.61 [1.20, 2.16]
Laifer et al., 2007	12	114	5	59	1.27 [0.43, 3.79]
Odone et al., 2011	620	1811	1059	3226	1.07 [0.94, 1.20]
Potter et al., 2020	1106	1901	126	336	2.32 [1.83, 2.94]
Langholz et al., 2019	852	1817	41	389	7.49 [5.35, 10.49]
Paulino et al., 2016	464	1673	906	3618	1.15 [1.01, 1.31]
Nordholm et al., 2022	1966	5489	496	3995	3.94 [3.53, 4.39]
Di Gennaro, 2023	115	290	53	218	2.05 [1.39, 3.02]
Riccardi, 2023	1988	5941	998	4614	1.82 [1.67, 1.99]
Willemstein, 2022	1466	2540	1693	4187	1.92 [1.74, 2.13]
Random Effects Model					2.07 [1.43, 3.00]

Heterogeneity: τ^2= 0.33, I^2=98.25%, H^2=57.1

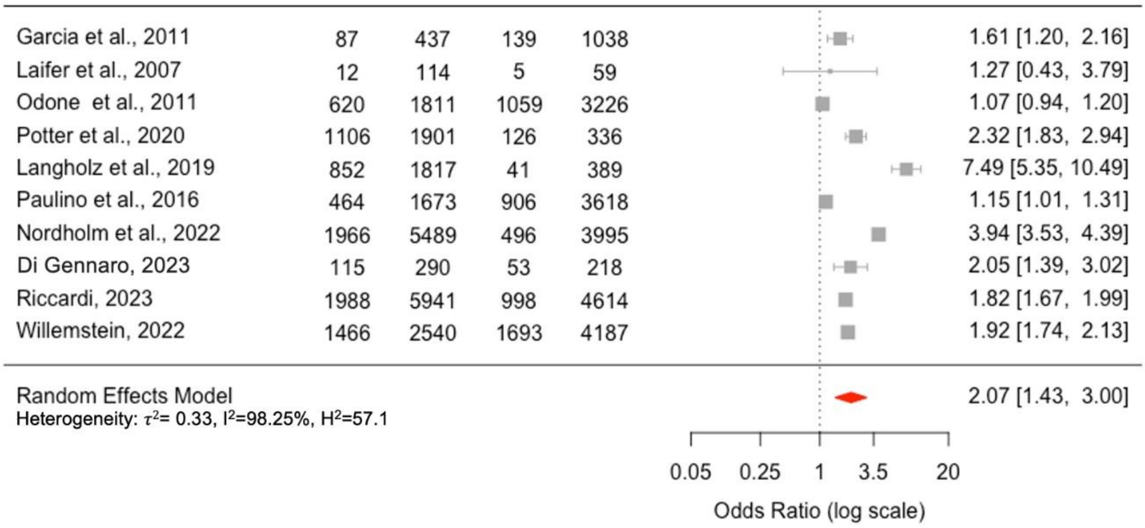

Supplement Figure S3 Quality assessment using the Newcastle-Ottawa Scale for risk of bias of studies included in the systematic review

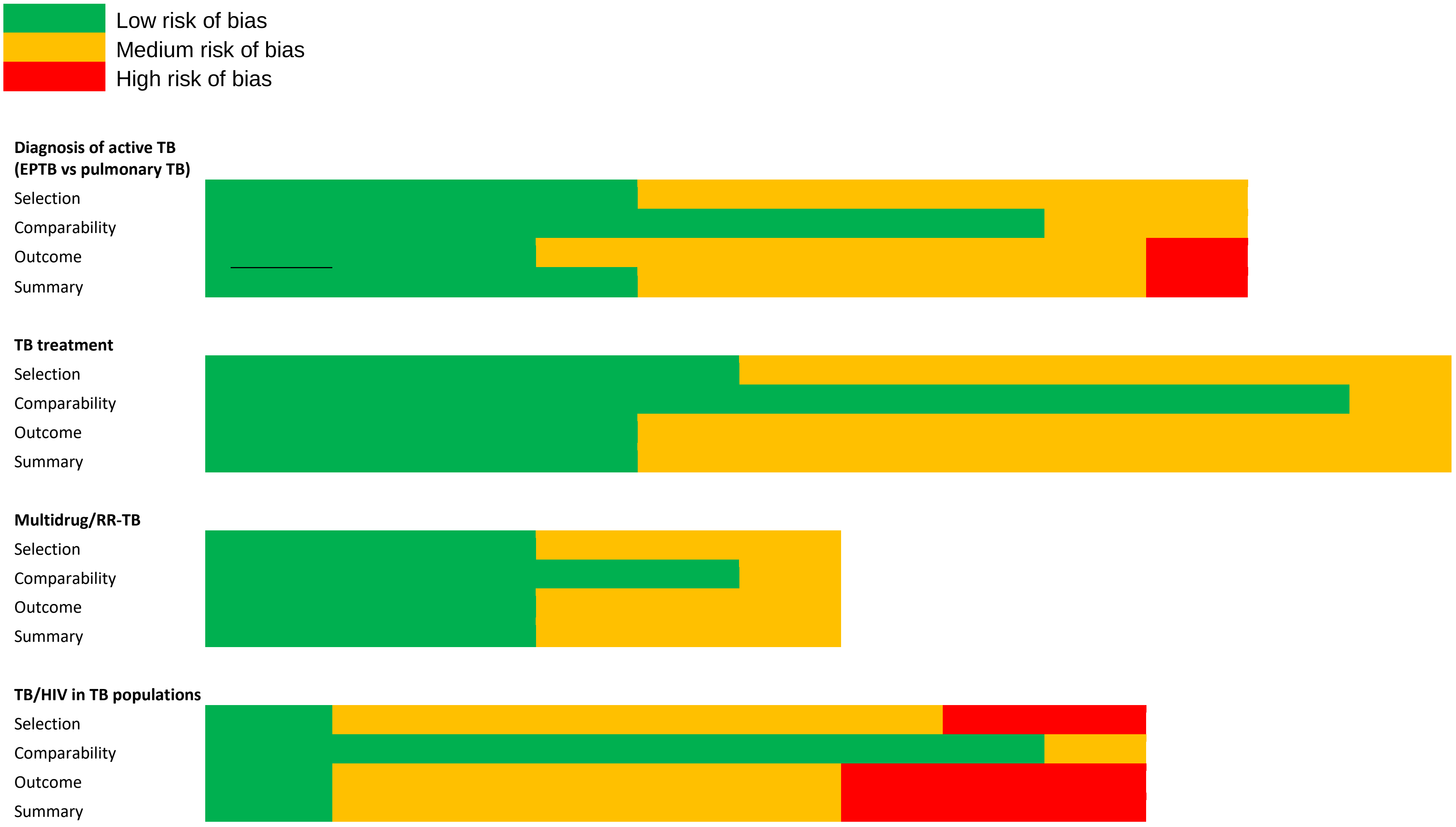

TB/HIV in HIV populations

Selection

Comparability

Outcome

Summary

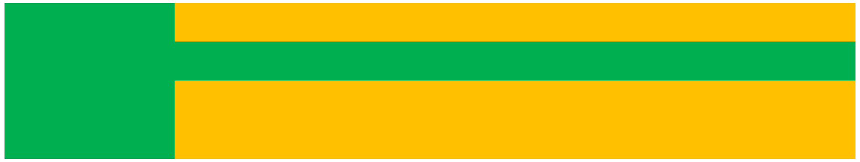

MDR/ RR TB=Multidrug-resistant/ Rifampicin resistant tuberculosis

EPTB = Extrapulmonary tuberculosis

Supplement table S2 PICOs for each expert chapter group

Chapter group	Content	Population	Intervention/ Exposure	Control	Outcome	Studies
**Diagnosis of active TB in migrants including the spectrum of clinical presentation**<br><br>**Delay in diagnosis**	Evaluation of migrants vs non-migrants in the diagnosis of active TB including the spectrum of clinical presentation and the delay in diagnosis	Migrants diagnosed with TB in clinical centers in the EU/EAA &UK	Diagnosis of active TB<br><br>Delay in diagnosis	Non-migrants diagnosed with TB in clinical centers in the EU/EAA &UK	Clinical presentation pulmonary vs extrapulmonary TB<br><br>Diagnostic delay including patient delay and health system delay	Observational cohort studies
**Treatment of TB in migrants including management issues**	Evaluation of the treatment of active TB in migrants vs non-migrants<br><br>Evaluation of management issues	Migrants treated for TB in clinical centers in the EU/EAA &UK	Treatment for drug-susceptible TB<br><br>Use of migrant-sensitive strategies	Non -migrants treated for TB in clinical centers in the EU/ EAA &UK<br><br>No use of migrant-sensitive strategies	TB treatment outcome	Observational cohort studies including non-controlled cohort studies Interventional studies.
**Treatment of MDR/RR-TB and management issues**	Evaluation of the diagnosis and management of MDR/RR TB in migrants vs non-migrants	Migrants treated for MDR/ RR TB in clinical centers in the EU/EAA &UK	Diagnosis of MDR/RR TB<br><br>Treatment for MDR/RR TB	Non -migrants treated for MDR/ RR TB in clinical centers in the EU/ EAA &UK<br><br>No use of migrant	Prevalence of MDR/RR TB<br><br>MDR/RR TB treatment outcomes	Observational cohort studies including non-controlled cohort studies Interventional studies.

			Use of migrant sensitive strategies	sensitive strategies		
**Special consideration for TB and HIV co-infection in migrants**	Evaluation of the diagnosis and management of TB/HIV coinfection in migrants vs non-migrants in the EU/EAA& UK	Migrants with TB/ HIV co-infection treated in clinical centers in the EU/EAA &UK	Diagnosis of HIV/TB co-infection<br><br>Treatment for TB	Non -Migrants with TB/ HIV co-infection treated in clinical centers in the EU/EAA&UK	Prevalence of TB/ HIV co-infection<br><br>TB treatment outcome	Observational cohort studies including non-controlled cohort studies Interventional studies.

Supplement table S3 Diagnosis of active tuberculosis (TB)-Extrapulmonary vs pulmonary TB studies

**Author Year Country**	**Study design Study period**<br><br>**Populations excluded**	**Migrants and non-migrants total**	**Migrants total**	**Non-migrants total**	**PTB in migrants**	**PTB in non-migrants**	**EPTB in migrants**	**EPTB in non-migrants**	**OR [95% CI]**
Paulino [1] 2016 Portugal	Retrospective National TB Surveillance study<br><br>2008-2012<br><br>Disseminated TB excluded	5291	1673	3618	1209	2712	464	906	1.15 [1.01, 1.31]
Nordholm [2]	Nationwide	9484	5489	3995	3523	3499	1966	496	3.94 [3.53, 4.391]

2022<br>Denmark	retrospective register-based case control study<br><br>1990-2018<br>.								
Garcia-Garcia [3]<br>2011<br>Spain	Prospective Multicentre cohort-study 2006-2007<br><br>Study period 2006-2007<br><br>Patients were included in extrapulmonary group if mixed features; unknown site was excluded	1475	437	1038	350	899	87	139	1.61 [1.20, 2.16]
Hayward [4]<br>2021<br><br>Multiple countries	Retrospective multi country Tessy database study<br><br>1995-2017	1270896	326987	943909	179173	739296	147814	204613	2.98 [2.96, 3.01]
Laifer [5]<br>2007<br>Switzerland	Retrospective hospital-based study	173	114	59	102	54	12	5	1.27 [0.43, 3.79]

	1997-2004<br><br>Denominator derived by percentage								
Odone [6]<br>2011<br>Italy	Retrospective regional TB surveillance system<br><br>1996-2006<br><br>Disseminated TB excluded	5037	1811	3226	1191	2167	620	1059	1.07 [0.94, 1.20]
Potter [7]<br>2020<br>UK	Retrospective national database study 2011-2016	2237	1901	336	795	210	1106	126	2.32 [1.83, 2.94]
Langholz [8]<br>2019<br>Denmark	Register-based prospective cohort study 1993-2015	2206	1817	389	965	348	852	41	7.49 [5.35, 10.49]
Di Gennaro [9]<br>Italy<br>2023	Retrospective cohort study 2013- 2021<br>Miliary TB excluded	508	290	218	175	165	115	53	2.05 [1.39-3.02]
Riccardi [10]	Retrospective	10555	5941	4614	3953	3616	1988	998	1.82 [1.67-1.99]

Italy<br>2023	cohort study<br><br>1990-2019<br><br>Pulmonary TB data obtained from author								
Willemstein [11]<br>The Netherlands<br>2022	Retrospective cohort study 2003 and 2018<br><br>Pulmonary and extrapulmonary group excluded	6727	2540	4187	1122	2494	1466	1693	1.92 [1.74-2.13]

Supplement table S4. Patient and health system delay studies

Author<br>Year<br>Country	Population<br>Study period	Study design	Total patients	Patient delay migrants<br>Median days (IQR)<br>Number of patients	Patient delay non-migrants<br>Median days (IQR)<br>Number of patients	Health system delay migrants<br>Median days (IQR)<br>Number of patients	Health system delay non-migrants<br>Median days (IQR)<br>Number of patients
Holden [12]<br>2019<br>Denmark	EPTB only<br>2009-2014	Retrospective national database study	N-450	60 (26-120)<br>N=336	30 (9-120)<br>N=61	28 (12-63)<br>N=358	38.5 (14-82)<br>N=70
Quattrocchi [13]<br>2018<br>Italy	EPTB and PTB<br>2014-2016	Prospective cross-sectional study	Patient delay 231<br>(IQR NA)<br>Health system delay 225 (IQR NA)	30 (14-60)<br>N=NA	15 (7-60)<br>N=NA	8 (4-22)<br>N=NA	21 (7.25-61)<br>N=NA
Zao [14]<br>2019<br>Portugal	EPTB and PTB<br>2010-2014<br><br>(Migrants 1162/<br>Non-migrants 5660)	National surveillance database study	NA	49 (NA)	32 (NA)	13 (NA)	18 9 (NA)

NA: not available

Supplement table S5 Tuberculosis treatment outcome studies

Author<br>Year<br>Country	Study design<br>Study period<br>Population	Definition of favourable treatment outcome	Definition of unfavourable treatment outcome	Total number of migrants	Total number of Non-migrants	Migrants favourable outcome	Migrants unfavourable Outcome	Non-migrants favourable outcome	Non-migrants Unfavourable Outcome	OR [95% CI]
Antoine [15]<br>2013<br>France	Retrospective<br>National database<br>2009	Treatment completed	Potentially unfavourable outcome	680	918	527	153	780	138	1.64 [1.27, 2.12]
Anyama [16]<br>2007<br>UK	Prospective<br>National Database Study<br>2001-2002	Successful treatment completion within 12 months	Treatment not completed or outcome unknown	266	189	215	51	143	46	0.74 [0.47, 1.16]
Borgdorff [17]<br>2000<br>Netherlands	Retrospective<br>National Database Study<br>1993-1997	Cure and treatment completion (Inclusive Definition used)	Treatment default	3916	3354	3447	469	3153	201	2.13 [1.80, 2.54]
Cayla[18]<br>2004<br>Spain	Prospective<br>Multicentre Cohort study<br>1999-2000	Cure and treatment completion	Treatment default	92	1114	76	16	1072	42	5.37 [2.89, 10.00]
Cayla [19]<br>2009<br>Spain<br>Percentage	Prospective<br>Multicentre Cohort Study<br>2006-2007	No treatment default, derived from percentage	Treatment default, there was no data on death, loss to follow up and unevaluated by	420	1004	369	51	963	41	3.25 [2.12, 4.98]

	Population derived from percentage		migrant status							
Cegolon [20] UK 2010	Retrospective London TB register-study 2003-2006	Cure and treatment completion	Treatment failure	8519	2136	7604	915	1906	230	1.00 [0.86, 1.16]
Garcia-Garcia [3] Spain 2011	Prospective Multicentre cohort-study 2006-2007	No treatment default	Unfavourable outcome = treatment default and unknown outcome	442	1048	369	73	963	85	2.24 [11.60, 3.13]
Holden [12] 2019 Denmark	Retrospective National Database Study 2009-2014<br><br>Extrapulmonary TB only	Treatment success and treatment completion	Unfavourable treatment outcome	330	67	313	17	64	3	1.16 [0.33, 4.07]
Karo [21] 2015 Norway/ Poland/ Romania/Slovenia/ UK	EU/ EAA Surveillance Database study 2002-2011	Treatment success	No treatment success = died, failed, default, transferred out and unknown outcome	37471	202129	29151	8320	159617	42512	1.07 [1.04, 1.10]
Mazza-Stalder[22] 2019 Switzerland	Retrospective Database study 2013-2014	Treatment success	Unsuccessful treatment outcome	288	87	262	26	76	11	0.69 [0.32, 1.45]

Paulino [1] Portugal 2016	Retrospective National TB Surveillance study 2008-2012	Treatment success	Treatment failure	2009	4111	1743	266	3581	530	1.03 [0.88, 1.21]
Di Gennaro [9] Italy 2023	Retrospective cohort study 2013- 2021	Treatment success	Unsuccessful treatment outcome= loss to follow up, death and failure	305	209	137	168	203	6	2.05 [1.39, 3.02]
Mechai [23] France 2022	Observational, prospective, multicenter cohort study 2018-2020.	Favourable treatment outcome	Unfavourable treatment outcome	123	22	100	23	14	8	0.4 [0.15, 1.07]
Riccardi [10] Italy 2023	Retrospective cohort study 1990-2019	Treatment success	Unfavourable treatment outcome = transferred out/ drop-out, died and failure	5941	4614	5358	583	4362	252	1.88 [1.62, 2.19]

Supplement table S6. Multidrug/rifampicin-resistant tuberculosis studies

Author<br>Year<br>Country	Study design<br>Study period	Number of total MDR/RR-TB patients	Number of MDR/RR-TB patients in migrants	Number of MDR/RR-TB patients in non-migrants	Percent of MDR cases in migrants
Bartu [24]<br>2010<br>Czech Republic	Retrospective cohort study<br>2001-2009	50	33	17	66
Bhering [25]<br>2019<br>Portugal	Retrospective cohort study<br>2000-2014	265	93	172	35.1
Flament - Saillour [26]<br>1999<br>France	Retrospective nationwide case-control study<br>1994	51	32	19	62.7
Fattorini [27]<br>2012<br>Italy	Retrospective laboratory study<br>2008-2010	198	162	36	81.8
Gavin [28]<br>2012<br>Spain	Retrospective laboratory study<br>1998-2008	478	202	276	42.3
Ghebremichael [29]<br>2008<br>Sweden	Retrospective laboratory study<br>1995- 2004	400	347	53	86.7
Helbling [30]<br>2014	Retrospective cohort study	51	46	5	90.2

Switzerland	2003-2010				
Ingen [31]<br>2008<br>Netherlands	Retrospective laboratory study<br>1993-2007	153	135	18	88.2
Ioannidis [32]<br>2017<br>Greece	Retrospective laboratory study<br>2007-2011	54	38	16	70.4
Kanavaki [33]<br>2006<br>Greece	Retrospective laboratory study<br>1993-2002	133	48	85	36.1
Oliveria [34]<br>2021<br>Portugal	Retrospective national database study<br>2000-2016	435	108	327	24.8
Seminari [35]<br>2020<br>Italy	Retrospective laboratory study<br>1998-2017	33	21	12	63.6
Van Altena[36]<br>2015<br>Netherlands	Retrospective cohort study<br>2000-2009	113	108	5	95.6
Vasankari [37]<br>2012<br>Finland	Retrospective cohort study<br>1994-2005	19	14	5	73.7
Vazquez-Gallardo [38]<br>2007<br>Spain	Prospective cohort study<br>1998 and 2004	58	7	51	12.1
Vluggen [39]	Retrospective laboratory	30	28	2	93.3

2017<br>Belgium	study<br>2010-2013				
Di Gennaro [9]<br>Italy<br>2023	Retrospective cohort study<br><br>2013- 2021<br>MDR TB = MDR TB and RR TB	77	71	6	96.8
Kherabi [40]<br>France<br>2022	Observational retrospective study<br><br>2008 to 2018	65	59	6	90.8

Supplement table S7. Multidrug/rifampicin-resistant vs drug-susceptible tuberculosis studies

Author<br>Year<br>Country	Study design<br>Study period	Number of total migrants	Number of total non-migrants	Number of MDR/RR-TB patients in migrants	Number of MDR/RR-TB patients in non-migrants	Number of patients with drug-susceptible TB in migrants	Number of patients with drug-susceptible TB in non-migrants	OR [95% CI]
Cannas [41]<br>2019<br>Italy	Prospective cohort study<br>2011-2016	651	206	42	9	609	197	1.51 [0.72, 3.16]
Gaborit [42]<br>2018<br>France	Retrospective cohort study<br>2002-2013	58	76	33	11	25	65	7.80 [3.42, 17.77]
Ferrara [43]<br>2005	Retrospective cohort study	1026	2353	39	88	987	2265	1.02 [0.69. 1.49]

Italy	1995-1999							
Glasauer [44] 2019 Germany	Retrospective national database study 2008-2017	12741	10126	478	70	12263	10056	5.60 [4.35, 7.21]
Jensenius [45] 2016 Norway	Retrospective national database study 1995-2014	36	894	85	4	3448	890	5.49 [2.01, 14.99]
Ruesen [46] 2014 Netherlands	Retrospective national database study 1993-2011	8914	4382	339	27	8575	4355	6.38 [4.30, 9.45]
Riccardi [10] Italy 2023	Retrospective cohort study<br><br>1990-2019<br><br>MDR TB =MDR and RR TB	4886	505	677	248	4209	2570	1.67 [1.43-1.94]

Supplement table S8. Tuberculosis/ HIV co-infection in Tuberculosis populations studies

Author<br>Year<br>Country	Study design<br>Study period	Total TB cases	Number of migrants	Number of non-migrants	Total TB HIV - co-infection	Total TB /HIV co-infection in migrants	Total TB /HIV co-infection in non-migrants	OR [95% CI]
Fronti [47]<br>2016<br>Italy	Observational retrospective cohort study<br>1998-2013	615	425	190	48	36	12	1.37 [0.70, 2.70]
Iñigo [48]<br>2006<br>Spain	Observational study<br>1994 - 2003	4263	472	3791	1391	62	1329	0.28 [0.21, 0.37]
Robert [49]<br>2000<br>France	Prospective study<br>1995-1997	2253	814	1439	246	89	157	1.00 [0.76, 1.32]
González-García [50]<br>2017<br>Spain	Observational retrospective study<br>1995-2013	1284	304	980	298	30	268	0.29 [0.19, 0.43]
Brindicci [51]<br>2016<br>Italy	Retrospective cohort study<br>2005-2013	129	44	85	11	3	8	0.70 [0.18, 2.80]
Diz Farina [52]<br>2007<br>Spain	Retrospective cohort study<br>1990-2002	1494	98	1397	532	16	516	0.33 [0.19, 0.58]

Paulino- [1]<br>2016<br>Portugal	Retrospective case-control study<br>2008-2012	6040	2009	4131	1123	452	671	1.50 [1.31, 1.71]
Meyssonnier [53]<br>2012<br>France	Retrospective cohort study<br>1995-2008	14610	7129	7481	1270	784	486	1.78 [1.58, 2.00]
Di Gennaro [9]<br>Italy<br>2023	Retrospective cohort study<br>2013- 2021	543	323	220	24	21	3	5.03 [1.48, 17.1]
Riccardi [10]<br>Italy<br>2023	Retrospective cohort study<br>1990-2019	10555	5941	4614	354	227	127	1.4 [1.13, 1.75]

Supplement table S9: TB/ HIV co-infection in people living with HIV studies

**Author**<br>**Year**<br>**Country**	**Study design**<br>**Study period**	**Total HIV cases**	**Number of Migrants**	**Number of Non-migrants**	**Total TB HIV - co-infection**	**Total TB/HIV co-infection in migrants**	**Total TB/HIV co-infection in non-migrants**	**OR [95% CI]**
Abgrall [54]<br>2010<br>France	Retrospective cohort study<br>1997-2008	72580	14491	58089	2625	1231	1394	3.78 [3.49, 4.09]
Camoni [55]<br>2013<br>Italy	Retrospective cohort study<br>1993-2010	45548	4867	40681	4075	1390	2685	5.66 [5.26, 6.09]
Velasco [56]<br>2008<br>Spain	Retrospective cohort study<br>1987-2006	7761	727	7034	1284	99	1185	0.78 [0.62. 0.97]
Martin-Iguacel [57]<br>2022<br>Denmark	Retrospective cohort study<br>1995–2017	6745	2020	4725	217	145	72	5.00 [3.75, 6.66]
Karo [58]<br>2014<br>Germany	Retrospective cohort study<br>2001-2011	11693	1403	8362	233	134	89	9.82 [7.46, 12.92]
Martin [59]<br>2011	Retrospective cohort study<br>1994–2005	3600	321	3279	1130	119	1011	1.32 [1.04, 1.68]

Spain								
Suarez [60] 2024 Germany	Retrospective cohort study 2006 -2017	4673	1290	3383	60	46	14	8.9 {4.87,16.24]

References

1. Paulino J, Martins A, Machado M, et al. Tuberculosis in native- and foreign-born populations in Portugal. *Int J Tuberc Lung Dis* 2016: 20(3): 357-362.

2. Nordholm AC, Andersen AB, Wejse C, et al . Social determinants of tuberculosis: a nationwide case-control study, Denmark, 1990-2018. *International journal of epidemiology* 2022: 51(5): 1446-1456.

3. Garcia-Garcia JM, Blanquer R, Rodrigo T, et al. Social, clinical and microbiological differential characteristics of tuberculosis among immigrants in Spain. *PloS one* 2011: 6(1): e16272.

4. Hayward SE, Rustage K, Nellums LB, et al. Extrapulmonary tuberculosis among migrants in Europe, 1995 to 2017. *Clin Microbiol Infect* 2021: 27(9): 1347.e1341-1347.e1347.

5. Laifer G, Widmer AF, Simcock M, et al. TB in a low-incidence country: differences between new immigrants, foreign-born residents and native residents. *The American journal of medicine* 2007: 120(4): 350-356.

6. Odone A, Ricco M, Morandi M,et al. Epidemiology of tuberculosis in a low-incidence Italian region with high immigration rates: differences between not Italy-born and Italy-born TB cases. *BMC public health* 2011: 11: 376.

7. Potter JL, Burman M, Tweed CD, et al. The NHS visitor and migrant cost recovery programme - a threat to health? *BMC public health* 2020: 20(1): 407.

8. Langholz Kristensen K, Lillebaek T, Holm Petersen J,.et al. Tuberculosis incidence among migrants according to migrant status: a cohort study, Denmark, 1993 to 2015. *Euro surveillance :* 2019: 24(44).

9. Di Gennaro F, Cotugno S, Fasano M, et al. High risk of unsuccessful treatment outcome in migrant population with tuberculosis: Data from three Italian hospitals. *Frontiers in public health* 2022: 10: 1024474.

10. Riccardi N, Antonello RM, Ferrarese M, et al. Tuberculosis in migrants: epidemiology, resistance and outcome in Milan, Italy. *Infectious diseases (London, England)* 2023: 55(8): 543-550.

11. Willemstein IJM, de Vries G, Essink DR, et al. TB in migrants residing in the Netherlands for at least 5 years at diagnosis, 2003-2018. *Int J Tuberc Lung Dis* 2022: 26(11): 1050-1057.

12. Holden IK, Lillebaek T, Andersen PH, et al. Extrapulmonary Tuberculosis in Denmark From 2009 to 2014; Characteristics and Predictors for Treatment Outcome. *Open forum infectious diseases* 2019: 6(10): ofz388.

13. Quattrocchi A, Barchitta M, Nobile CGA, et al. Determinants of patient and health system delay among Italian and foreign-born patients with pulmonary tuberculosis: a multicentre cross-sectional study. *BMJ open* 2018: 8(8): e019673.

14. Zao I, Ribeiro AI, Apolinario D, et al. Why does it take so long? The reasons behind tuberculosis treatment delay in Portugal. *Pulmonology* 2019: 25(4): 215-222.

15. Antoine D, Che D. Treatment outcome monitoring of pulmonary tuberculosis cases notified in France in 2009. *Euro Surveill* 2013: 18(12).

16. Anyama N, Bracebridge S, Black C, et al. What happens to people diagnosed with tuberculosis? A population-based cohort. *Epidemiology and Infection* 2007: 135(7): 1069-1076.

17. Borgdorff MW, Veen J, Kalisvaart NA, et al. Defaulting from tuberculosis treatment in The Netherlands: rates, risk factors and trend in the period 1993-1997. *The European respiratory journal* 2000: 16(2): 209-213.

18. Cayla JA, Caminero JA, Rey R, et al. Current status of treatment completion and fatality among tuberculosis patients in Spain. *Int J Tuberc Lung Dis* 2004: 8(4): 458-464.

19. Cayla JA, Rodrigo T, Ruiz-Manzano J, et al. Tuberculosis treatment adherence and fatality in Spain. *Respiratory research* 2009: 10: 121.

20. Cegolon L, Maguire H, Mastrangelo G, et al. Predictors of failure to complete tuberculosis treatment in London, 2003-2006. *Int J Tuberc Lung Dis* 2010: 14(11): 1411-1417.

21. Karo B, Hauer B, Hollo V, et al. Tuberculosis treatment outcome in the European Union and European Economic Area: an analysis of surveillance data from 2002-2011. *Eurosurveillance* 2015: 20(49).

22. Mazza-Stalder J, Chevallier E, Opota O, et al. Improvement in Tuberculosis Outcomes With a Combined Medical and Social Approach. *Frontiers in medicine* 2019: 6: 135.

23. Mechai F, Bachelet D, Han L, et al. Tuberculosis treatment outcomes among precarious patients in France. *Infectious diseases now* 2022: 52(7): 389-395.

24. Bartu V, Kopecka E, Havelkova M. Factors associated with multidrug-resistant tuberculosis: comparison of patients born inside and outside of the Czech Republic. *The Journal of international medical research* 2010: 38(3): 1156-1163.

25. Bhering M, Kritski A, Nunes C, et al. Multidrug-resistant tuberculosis in Lisbon: unfavourable treatment and associated factors, 2000-2014. *Int J Tuberc Lung Dis* 2019: 23(10): 1075-1081.

26. Flament-Saillour M, Robert J, Jarlier V, et al. Outcome of multi-drug-resistant tuberculosis in France: a nationwide case-control study. *Am J Respir Crit Care Med* 1999: 160(2): 587-593.

27. Fattorini L, Mustazzolu A, Piccaro G, et al. Drug-resistant tuberculosis among foreign-born persons in Italy. *The European respiratory journal* 2012: 40(2): 497-500.

28. Gavin P, Iglesias MJ, Jimenez MS, et al. Long-term molecular surveillance of multidrug-resistant tuberculosis in Spain. *Journal of molecular epidemiology and evolutionary genetics in infectious diseases* 2012: 12(4): 701-710.

29. Ghebremichael S, Petersson R, Koivula T, et al. Molecular epidemiology of drug-resistant tuberculosis in Sweden. *Microbes and infection* 2008: 10(6): 699-705.

30. Helbling P, Altpeter E, Egger JM, et al. Treatment outcomes of multidrug-resistant tuberculosis in Switzerland. *Swiss medical weekly* 2014: 144: w14053.

31. Ingen Jv, Boeree MJ, Wright A, Laan Tvd, et al. Second-line drug resistance in multidrug-resistant tuberculosis cases of various origins in the Netherlands. *Int J Tuberc Lung Dis* 2008: 12(11): 1295-1299.

32. Ioannidis P, van Soolingen D, Mokrousov I, et al. Multidrug-resistant/extensively drug-resistant tuberculosis in Greece: predominance of Mycobacterium tuberculosis genotypes endemic in the Former Soviet Union countries. *Clin Microbiol Infect* 2017: 23(12): 1002-1004.

33. Kanavaki S, Mantadakis E, Nikolaou S, et al. Resistance of Mycobacterium tuberculosis isolates in different populations in Greece during 1993-2002. *Int J Tuberc Lung Dis* 2006: 10(5): 559-564.

34. Oliveira O, Gaio R, Correia-Neves M, et al. Evaluation of drug-resistant tuberculosis treatment outcome in Portugal, 2000-2016. *PloS one* 2021: 16(4): e0250028.

35. Seminari E, Monzillo V, Lombardi A, et al. Migrations do not modify Mycobacterium tuberculosis resistance rates: a 20-year retrospective study. *European journal of clinical microbiology & infectious diseases* 2020: 39(6): 1083-1087.

36. Van Altena R, De Vries G, Haar CH, et al. Highly successful treatment outcome of multidrug-resistant tuberculosis in the Netherlands, 2000-2009. *Int J Tuberc Lung Dis* 2015: 19(4): 406-412.

37. Vasankari T, Soini H, Liippo K, et al. MDR-TB in Finland--still rare despite the situation in our neighbouring countries. *The clinical respiratory journal* 2012: 6(1): 35-39.

38. Vazquez-Gallardo R, Anibarro L, Fernandez-Villar A, et al. Multidrug-resistant tuberculosis in a low-incidence region shows a high rate of transmission. *Int J Tuberc Lung Dis* 2007: 11(4): 429-435.

39. Vluggen C, Soetaert K, Groenen G, et al. Molecular epidemiology of Mycobacterium tuberculosis complex in Brussels, 2010-2013. *PloS one* 2017: 12(2): e0172554.

40. Kherabi Y, Mollo B, Gerard S, et al. Patient-centered approach to the management of drug-resistant tuberculosis in France: How far off the mark are we? *PLOS global public health* 2022: 2(4): e0000313.

41. Cannas A, Butera O, Gualano G, et al. Multidrug-Resistant Tuberculosis In A Referral Center In Rome: 2011- 2016. *Infection and drug resistance* 2019: 12: 3275-3281.

42. Gaborit BJ, Revest M, Roblot F, et al. Characteristics and outcome of multidrug-resistant tuberculosis in a low-incidence area. *Medecine et maladies infectieuses* 2018: 48(7): 457-464.

43. Ferrara G, Richeldi L, Bugiani M, et al. Management of multidrug-resistant tuberculosis in Italy. *Int J Tuberc Lung Dis* 2005: 9(5): 507-513.

44. Glasauer S, Altmann D, Hauer B, et al. First-line tuberculosis drug resistance patterns and associated risk factors in Germany, 2008-2017. *PloS one* 2019: 14(6): e0217597.

45. Jensenius M, Winje BA, Blomberg B, et al. Multidrug-resistant tuberculosis in Norway: a nationwide study, 1995-2014. *Int J Tuberc Lung Dis* 2016: 20(6): 786-792.

46. Ruesen C, Gageldonk-Lafeber ABv, et al. Extent and origin of resistance to antituberculosis drugs in the Netherlands, 1993 to 2011. *Eurosurveillance* 2014: 19(11).

47. Fronti E, Vecchia M, Scudeller L, et al. Epidemiology of Mycobacterium tuberculosis infection in Pavia province, Lombardy, Northern Italy, 1998-2013. *The new microbiologica* 2016: 39(4): 264-268.

48. Inigo J, Arce A, Rodriguez E, et al. Tuberculosis trends in Madrid, 1994-2003: impact of immigration and HIV infection. *Int J Tuberc Lung Dis* 2006: 10(5): 550-553.

49. Robert J, Trystram D, Truffot-Pernot C, et al. Surveillance of Mycobacterium tuberculosis drug resistance in France, 1995-1997. *Int J Tuberc Lung Dis* 2000: 4(7): 665-672.

50. Gonzalez-Garcia A, Fortun J, Elorza Navas E, et al. The changing epidemiology of tuberculosis in a Spanish tertiary hospital (1995-2013). *Medicine* 2017: 96(26): e7219.

51. Brindicci G, Santoro CR, Trillo G, et al. Prevalence and Clinical Characteristics of Mycobacterial Diseases in the Barletta-Andria-Trani Province, Italy (2005-2013). *BioMed research international* 2016: 2016: 9362708.

52. Diz Farina S, Lopez-Velez R, Moreno A, et al. Epidemiology and clinical features of tuberculosis in immigrants at an infectious diseases department in Madrid. *Int J Tuberc Lung Dis* 2007: 11(7): 769-774.

53. Meyssonnier V, Veziris N, Bastian S, et al. Increase in primary drug resistance of Mycobacterium tuberculosis in younger birth cohorts in France. *J Infect* 2012: 64(6): 589-595.

54. Abgrall S, Del Giudice P, Melica G, et al. HIV-associated tuberculosis and immigration in a high-income country: incidence trends and risk factors in recent years. *AIDS* 2010: 24(5): 763-771.

55. Camoni L, Regine V, Boros S, et al. AIDS patients with tuberculosis: characteristics and trend of cases reported to the National AIDS Registry in Italy--1993-2010. *European journal of public health* 2013: 23(4): 658-663.

56. Velasco M, Castilla V, Cervero M, et al. The changing pattern of tuberculosis and HIV co-infection in immigrants and Spaniards in the last 20 years. *HIV medicine* 2008: 9(4): 227-233.

57. Martin-Iguacel R, Llibre JM, Pedersen C, et al. Tuberculosis incidence and mortality in people living with human immunodeficiency virus: a Danish nationwide cohort study. *Clinical microbiology and infection* 2022: 28(4): 570-579.

58. Karo B, Haas W, Kollan C, et al. Tuberculosis among people living with HIV/AIDS in the German ClinSurv HIV Cohort: long-term incidence and risk factors. *BMC Infect Dis* 2014: 14: 148.

59. Martin V, de Olalla PG, Orcau A, et al. Factors associated with tuberculosis as an AIDS-defining disease in an immigration setting. *Journal of Epidemiology* 2011: 21(2): 108-113.

60. Suarez I, Rauschning D, Schuller C, et al. Incidence and risk factors for HIV-tuberculosis coinfection in the Cologne-Bonn region: a retrospective cohort study. *Infection* 2024.
